# Adrenergic receptor blockade attenuates placental ischemia‐induced hypertension

**DOI:** 10.14814/phy2.13814

**Published:** 2018-09-03

**Authors:** Frank T. Spradley, Ying Ge, B. Peyton Haynes, Joey P. Granger, Christopher D. Anderson

**Affiliations:** ^1^ Department of Surgery The University of Mississippi Medical Center Jackson Mississippi; ^2^ Department of Physiology & Biophysics The University of Mississippi Medical Center Jackson Mississippi; ^3^ Cardiovascular‐Renal Research Center The University of Mississippi Medical Center Jackson Mississippi; ^4^ Women's Health Research Center The University of Mississippi Medical Center Jackson Mississippi

**Keywords:** Placenta, PlGF, preeclampsia, rat, RUPP

## Abstract

Preeclampsia (PE), a disorder of new‐onset maternal hypertension and vascular dysfunction during pregnancy, is thought to be linked to placental ischemia‐induced release of prohypertensive factors and reductions of vasoprotective factors in the maternal circulation. Although markers of sympathetic nervous activity are elevated in experimental models of placental ischemia‐induced hypertension and women with PE compared with their normal pregnant counterparts, the importance of adrenergic receptor signaling in the development of hypertension in PE is unknown. Therefore, we tested the hypothesis that adrenergic receptor blockade attenuates the development of placental ischemia‐induced hypertension in rats. Wistar Hannover rats underwent reduced uterine perfusion pressure (RUPP) or Sham surgeries on gestational day 14. By day 19, mean arterial blood pressure (MAP) was increased in RUPP over Sham rats. Groups of RUPP and Sham pregnant rats received terazosin and propranolol (3 mg/kg per day of each via subcutaneous osmotic minipump) to block *α*_1_‐ and *β*‐adrenergic receptors, respectively, beginning on gestational day 14. Adrenergic blockade significantly attenuated the development of hypertension in the RUPP rats with a slight blood pressure‐lowering response in the Sham, normal pregnant rats by day 19. In conclusion, these data implicate that placental ischemia‐induced hypertension involves adrenergic receptor signaling to promote increases in blood pressure during PE.

## Introduction

Hypertensive disorders of pregnancy and maternal cardiovascular diseases are the number one cause of pregnancy‐related deaths in the United States (Creanga et al. 2017). These disorders include chronic hypertension, gestational hypertension, preeclampsia (PE), and chronic hypertension with superimposed PE (American College of Obstetricians, Gynecologists, and Task Force on Hypertension in P., 2013). PE is clinically defined as new‐onset hypertension occurring during the second half of pregnancy along with additional systemic vascular abnormalities in the mother. These include reduced cardiac reserve, cerebral vascular autoregulation, and renal hemodynamics along with increased glomerular injury, pulmonary edema, and cerebral edema (Stillman and Karumanchi 2007; American College of Obstetricians , Gynecologists, and Task Force on Hypertension in Pregnancy, 2013; Gandhi et al. 2014; Melchiorre et al. 2014; Hammer and Cipolla 2015; von Dadelszen and Magee 2016; Logue et al. 2016). Complications of PE appear to be more diverse than other hypertensive disorders of pregnancy with dysfunction in organ systems like the heart, kidney, liver, brain, and placenta (Shen et al. 2017; Tooher et al. 2017). PE increases the risk for preterm birth and intrauterine growth restriction (IUGR) in the fetus (Srinivas et al. 2009; Parker and Werler 2014; Sharma et al. 2016), and these offspring are predisposed to cardiometabolic diseases as they age (Spradley et al. 2018). PE impacts 2–8% of pregnancies per year in the USA with a prior preeclamptic pregnancy significantly increasing the risk for PE in subsequent pregnancies (Roberts et al. 2011; Bartsch et al. 2016). Epidemiological findings highlight that the rates of PE are rising (Knight 2008; Roberts et al. 2011; Ananth et al. 2013; Breathett et al. 2014). Unfortunately, the most effective “cure” is delivery of the fetus and ischemic placenta (Townsend et al. 2016). The ischemic placenta is hypothesized to play a causative role in the pathogenesis of PE by eliciting the release of soluble prohypertensive factors and reducing vasoprotective factors like the angiogenic factor, placental growth factor (PlGF), into the maternal circulation thereby driving maternal vascular dysfunction and hypertension (Granger et al. 2018).

While a major focus of study in the mechanisms behind PE has been on the link between placental factors and vascular dysfunction, several studies have implicated the sympathetic division of the autonomic nervous system in the pathogenesis of PE. Schobel et al. (1996) assessed multiunit recordings of postganglionic sympathetic nervous system activity (SNA) of the peroneal nerve to quantitate muscle (M)SNA and observed that MSNA is elevated in normotensive pregnant women compared to nonpregnant, normotensive counterparts, which is a repeated finding (Jarvis et al. 2012; Charkoudian et al. 2017; Reyes et al. 2018b; Schmidt et al. 2018). Reyes et al. (2018a) reviewed studies demonstrating that MSNA is elevated in women with PE over normal pregnant and hypertensive, nonpregnant control women. Furthermore, there is evidence of greater adrenergic receptor signaling, whereby the blood pressure response to acute infusions of norepinephrine is greater during pregnancy‐induced hypertension (Raab 1957; Zuspan et al. 1964; Talledo et al. 1968; Nisell et al. 1985a). Women with PE have antihypertensive responses to nonselective adrenergic receptor blocker administration (Cleary et al. 2018). However, this administration protocol occurs after the onset of hypertension. Therefore, it is unknown if this receptor system plays a permissive role or actively mediates the development of hypertension in PE.

Although there is a body of literature demonstrating an association between increases in SNA and PE, there are no published intervention studies examining the causative role for adrenergic receptor signaling in mediating the hypertension during PE. This highlights the need for experimental models of PE to address this topic. Studies using such models support that SNA and vasoconstriction to adrenergic agonists are increased in PE (Costantine et al. 2010; Intapad et al. 2014; Makris et al. 2016; Spradley et al. 2016). Placental ischemia‐induced hypertension in the reduced uterine perfusion pressure (RUPP) rat model is associated with a hypertensive shift in baroreceptor control on renal SNA (Hines et al. 2007). Furthermore, the hypertension in various models of PE, such as RUPP or excess of placental ischemic factors, is associated with greater adrenergic receptor‐induced vasoconstriction (Costantine et al. 2010; Brennan et al. 2016; Zhu et al. 2016). Although experimental models and humans with PE have elevated markers of SNA and adrenergic receptor signaling, the importance of this system in the development of placental ischemia‐induced hypertension has yet to be investigated. Therefore, we tested the hypothesis that adrenergic receptor blockade attenuates the development of placental ischemia‐induced hypertension in rats. We initiated adrenergic receptor blockade on the day of RUPP surgery to determine if intervening in this system prevents the hypertensive response or impacts circulating levels of PlGF.

## Material and Methods

### Animals

All animal experiments were conducted in accordance with the National Institutes of Health *Guide for the Care and Use of Laboratory Animals*. The University of Mississippi Medical Center's Institutional Animal Care and Use Committee approved all experimental animal protocols. Timed‐pregnant Wistar Hannover (WH) rats were generated utilizing WH males from an in‐house, outbred colony at our institution. The colony and experimental rats were maintained on Teklad 8640 standard chow diet (Envigo; Madison, WI). Gestational day (GD) 0 was defined as the presence of sperm in the vaginal smear. All surgeries and tissue harvests were conducted in rats under isoflurane anesthesia (Butler Schein Animal Health, Dublin, OH) using aseptic techniques.

### Chronic protocols for RUPP and adrenergic receptor blockade

On GD 14, the RUPP procedure was performed, as previously described (Alexander et al. 2001). Briefly, the RUPP surgery involves placement of a silver clip (0.203 mm, internal diameter) on the subrenal abdominal aorta above the uterine arteries along with clips (0.1 mm, internal diameter) on branches of the ovarian artery in both uterine horns. The control group consisted of a Sham surgery with similar abdominal incision and suturing.

Adrenergic receptor blockers were administered into additional Sham and RUPP rats. For this purpose, Alzet osmotic minipumps (model 2ML1, Durect Corp; Cupertino, CA) were loaded with 3 mg/kg per day each of terasozin HCl and propranolol HCl (Millipore Sigma, St. Louis, MO) diluted in sterile 0.9% saline (Baxter; Deerfield, IL). Terasozin is a blocker of *α*_1_‐adrenergic receptors and propranolol blocks *β*_1_‐ and *β*_2_‐adrenergic receptors. Pumps were placed dorsally for subcutaneous infusion and closed with staples on GD 14 at the time of the RUPP or Sham RUPP procedures. Sham pump surgeries were conducted in the untreated groups.

### Maternal blood pressure measurements

On GD 18, indwelling catheters were implanted in the left carotid artery and exposed at the nape of the neck. Catheters consisted of V/1 tubing attached to V/3 tubing (Scientific Commodities, Lake Havasu City, AZ). Approximately, 2.5 cm of the V/3 end of the catheter was inserted into the carotid. Catheters were filled with sterile heparin‐0.9% saline solution (300 mg/mL; Pfizer, New York City, NY) and stoppered with a stainless‐steel catheter plug (SP22/12; Instech Laboratories, Plymouth Meeting, PA) to maintain patency. On GD 19, conscious mean arterial blood pressure and heart rates were measured, as described in previous publications (Alexander 2003; Banek et al. 2013; Murphy et al. 2013; Spradley et al. 2013). For this purpose, rats were placed in restrainers (Kent Scientific Corp, Torrington, CT) and catheters connected to pressure transducers (MLT0699; ADInstruments, Colorado Springs, CO) coupled to a computerized data acquisition system (PowerLab, ADInstruments). Readings were calibrated on every rat then data acquired at 1 k/sec. Once blood pressure readings stabilized (~1 h), ~10 min of mean arterial blood pressure (MAP) and heart rate data were collected and averaged. The % fall of MAP in response to adrenergic blockade treatment was calculated for each Sham or RUPP group by: % fall = ((individual treated MAP ‐ average untreated MAP)/(average untreated MAP))*100.

### Blood collection and pregnancy biometrics

On GD 19, a midline incision was made and uterine horns with fetuses exteriorized. Blood was collected from the abdominal aorta into Vacutainer K_2_EDTA tubes (BD, Franklin Lakes, NJ), spun at 644 *g* for 12 min at 4°C, and plasma stored at −20°C. It was ensured that each fetus and matching placenta were weighed and recorded as individual fetal‐placental units. Average fetal and placental weights were calculated per rat then averaged for each experimental group. Total viable or resorbed fetuses were noted. Percent fetal resorption = (number of resorbed fetuses/total number of fetuses)*100. Placental sufficiency = average fetal weight/average placental weight for each dam, as a surrogate measure of the nurturing capacity of the placenta, as previously described in humans (Hunt et al. 2016).

### Vasoconstriction studies

Isolated third‐order mesenteric arteries were cleaned of perivascular adipose tissue and renal interlobar arteries gently dissected and cleared of renal tissue for vascular function studies. Vascular rings of ~2.5 mm in length were mounted on chucks in a wire myograph (model 620M, Danish Myo Technology A/S, Aarhus, Denmark) containing 5 mL PSS (concentration in mmol/L: 118.3 NaCl, 4.7 KCl, 2.5 CaCl_2_, 1.2 MgSO_4_, 1.2 KH_2_PO_4_, 25 NaHCO_3_, and 11.1 dextrose) warmed to 37°C and bubbled with carbogen. Four mN of preload was placed on the arterial rings. For mesenteric arteries, blood vessel integrity following the isolation procedure was examined with a dose of phenylephrine (Phe at ~1E^−5^ M, Sigma) to produce vasoconstriction followed by a dose of acetylcholine (Ach at 2E^−3^ M, Sigma) to ensure vasorelaxation. As for interlobar arteries, rings were constricted with Phe (4E^−7^ M) and their integrity confirmed by vasorelaxation to adenosine diphosphate (ADP at 40E^−5^ M, RPI Corp; Mt Prospect, IL). Arterial segments were then washed with PSS, equilibrated for 15 min, and cumulative concentration–response curves were generated to increasing concentrations of Phe (1E^−9^–3E^−4^ M) and KCl (8–133 mmol/L) (Fisher). Phe responses were normalized and graphed as a % of maximum KCl‐induced vasoconstriction. KCl responses are graphed as % increase in force from baseline. An *N* = 1 was defined as 1 ring/rat.

### Quantification of circulating PlGF

Plasma levels of PlGF were quantified using a Quantikine enzyme‐linked immunosorbent assay (ELISA) kit from R&D Systems (Minneapolis, MN) detecting mouse PlGF‐2 (MP200). Utilizing NCBI Protein Basic Local Alignment Search Tool (BLAST) software, it was found that mouse and rat PlGF‐2 have 91% alignment (https://blast.ncbi.nlm.nih.gov/Blast.cgi).

### Statistical analysis

Data were graphed and analyzed using GraphPad Prism version 7.0 (La Jolla, CA). Data are presented as mean ± standard error of the mean (SEM). Statistically significant differences were defined as *P* < 0.05. Two‐way analysis of variance (ANOVA) tests were conducted followed by Tukey's multiple comparisons tests. In the data described for Figure 4, the effective maximum response (*E*_max_) and total response (area under the curve, AUC) in the vascular reactivity studies were analyzed by Student's *t*‐tests. In addition, a two‐way analysis of variance with repeated measures followed by Sidak's multiple comparisons test was used to assess differences for each concentration of the vasoconstriction curves.

## Results

### Chronic adrenergic receptor blockade on maternal blood pressure

**Figure 1:**
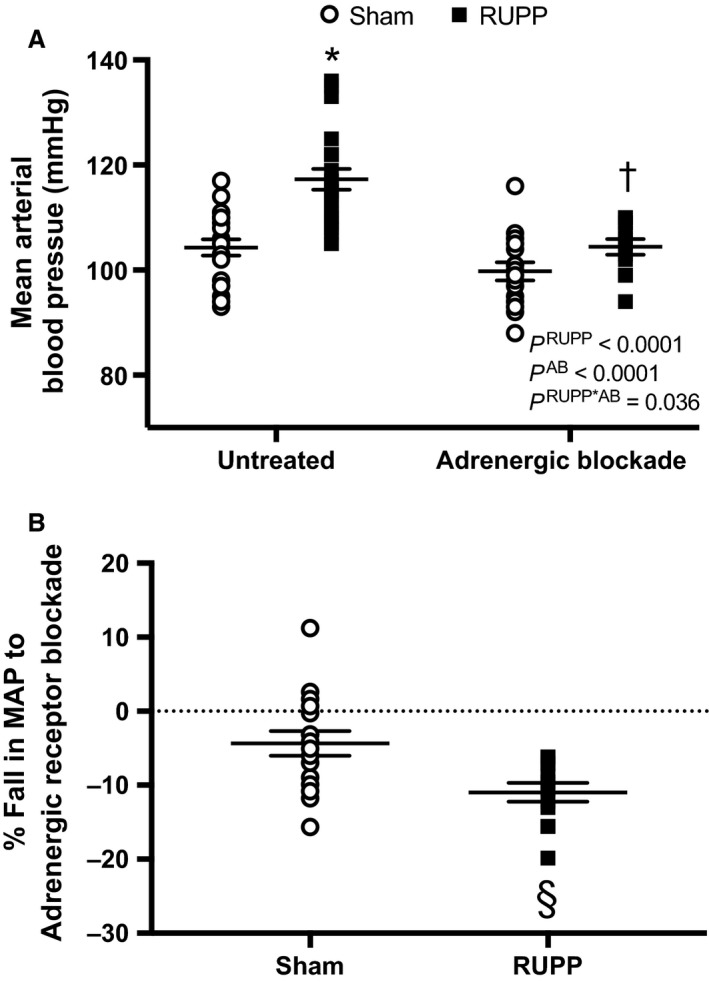
Sham or RUPP rats were either untreated (*N* = 20 and 26, respectively) or treated with adrenergic receptor blockade (AB) (*N* = 16 and 12, respectively) from GD 14–19. (A) Mean arterial blood pressure (MAP). Inset: results from the two‐way ANOVA. According to Tukey's multiple comparisons test: *P < 0.0001 for untreated RUPP versus untreated Sham, ^†^ *P* = 0.0001 for untreated RUPP versus adrenergic receptor blocker‐treated RUPP. (B) the % fall in MAP in response to adrenergic blockade treatment. Assessment by an unpaired *t* test revealed ^§^ *P* = 0.006.

Figure 1A illustrates the impact of chronic adrenergic receptor blockade on blood pressure in RUPP and Sham rats. Blood pressure was higher in RUPP rats and adrenergic receptor blockade reduced blood pressure in both RUPP and Sham groups. Importantly, there was a statistically significant interaction between the RUPP and Sham pregnant groups and the treatment blockade groups indicating greater adrenergic receptor‐mediated blockade of hypertension in RUPP rats. This is further demonstrated in Figure 1B, where the % fall in blood pressure response to adrenergic blockade was greater in RUPP rats. Indeed, there was a fall in blood pressure in response to blockade of −10 ± 1 mmHg for RUPP rats, whereas it was only −4 ± 2 mmHg in the Sham group. This difference was significantly greater in the former.

Heart rate was similar in untreated RUPP and Sham groups (426 ± 5 vs. 398 ± 20 bpm, *P* = 0.93), respectively. Overall, heart rate was significantly (*P* = 0.004) lower in response to adrenergic receptor blockade in Sham (389 ± 7 bpm) and RUPP (363 ± 16 bpm) rats, with the interaction also being significant (*P* = 0.038), but the post hoc test did not reveal any specific differences in heart rate for the effect of adrenergic receptor blockade between Sham or RUPP rats.

### Fetal and placental biometrics

**Figure 2:**
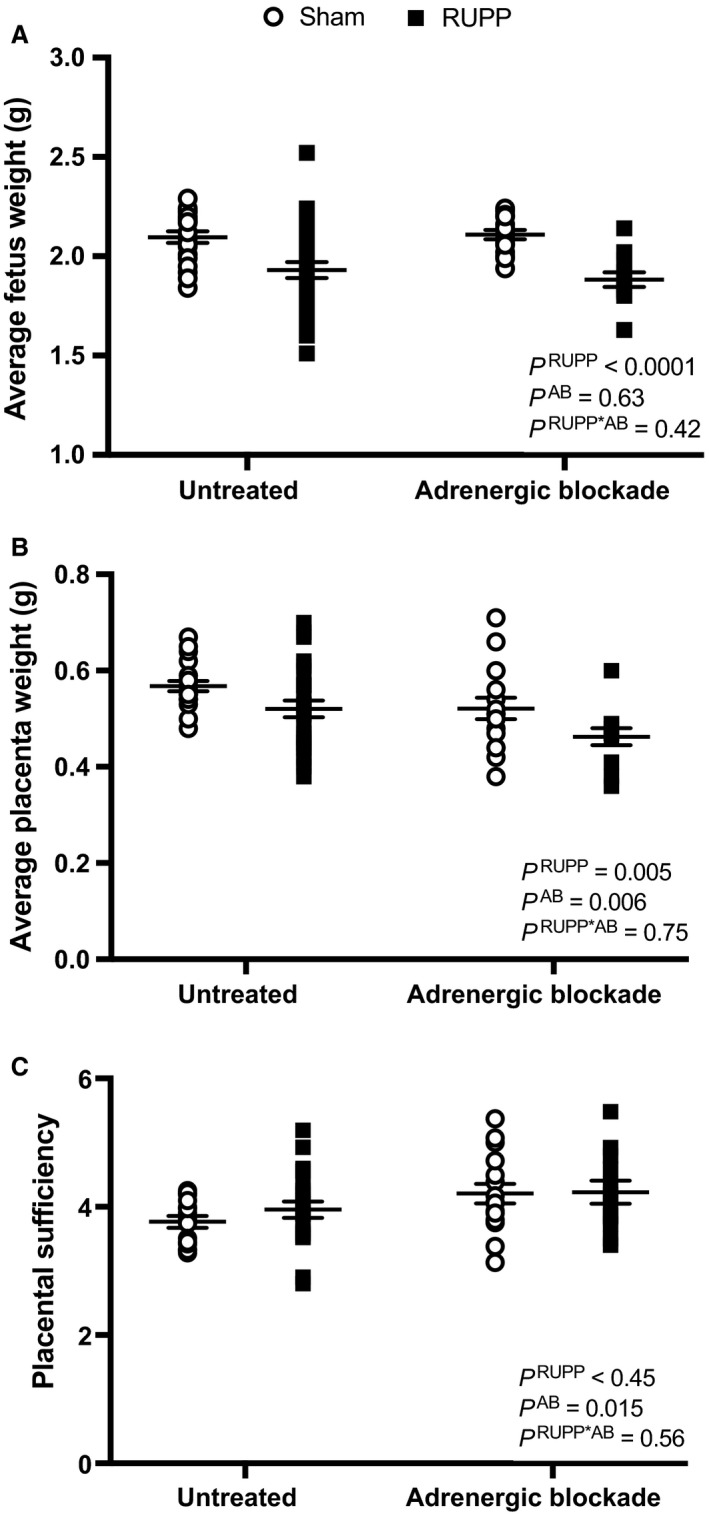
Average fetus weights (A), average placenta weights (B), and placental sufficiency (C) assessed at GD 19 in Sham or RUPP rats that were either untreated (*N* = 20 and 26, respectively) or treated with adrenergic receptor blockade (AB) (*N* = 16 and 12, respectively). Inset: results from two‐way ANOVAs.

Figure 2A depicts average fetal weights in Sham and RUPP groups in the presence or absence of adrenergic receptor blockade. RUPP reduced fetal weights, but adrenergic receptor blockade did not alter this parameter in RUPP or Sham groups. The values for % fetal resorptions were: 3 ± 2 (Sham untreated), 7 ± 2 (Sham treated), 58 ± 4 (RUPP untreated), and 70 ± 4 (RUPP treated). The % number of fetal resorptions was greater in the RUPP groups and also in the blockade groups but with no statistical interaction between these two factors. With regards to average placental weights, this value was reduced in RUPP groups and adrenergic receptor blockade groups without a statistical interaction (Fig. 2B). Placental sufficiency, as a measure of the ability of the placenta to nurture the fetus, was not altered by the RUPP procedure but was overall greater in the adrenergic receptor blockade treatment groups with no statistical interaction (Fig. 2C).

### Circulating PlGF

**Figure 3:**
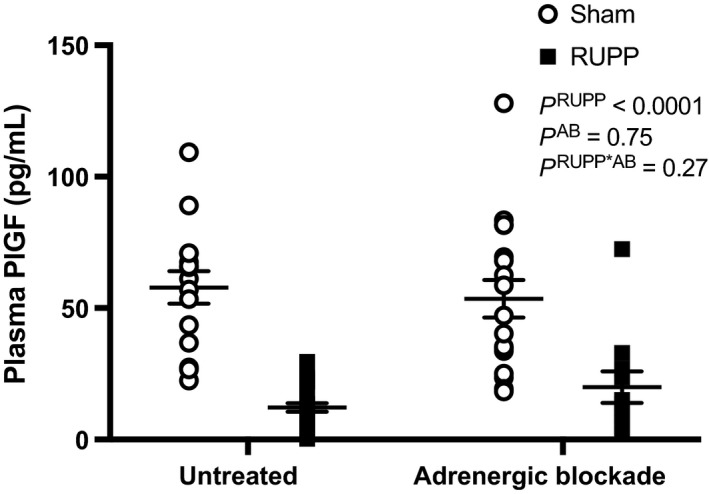
Plasma levels of placental growth factor (PlGF) at GD 19 from Sham or RUPP rats were either untreated (*N* = 15 and 23, respectively) or treated with adrenergic receptor blockade (AB) (*N* = 17 and 11, respectively) from GD 14–19. Inset: results from the two‐way ANOVA.

Figure 3 shows that plasma PlGF levels were significantly reduced in RUPP compared with Sham pregnant rats. These levels were not impacted by treatment with adrenergic receptor blockers in either Sham or RUPP groups.

### Impact of RUPP on adrenergic receptor‐induced vasoconstriction

**Figure 4:**
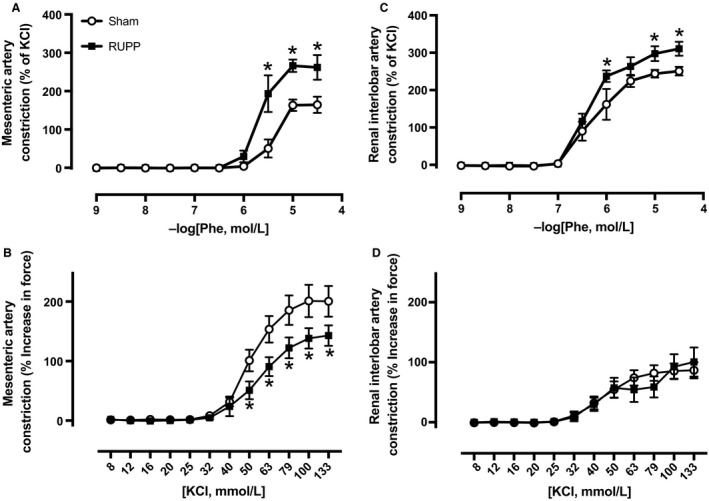
Vasoconstriction to cumulative concentrations of phenylephrine or KCl in third‐order mesenteric arteries (A, B) and renal interlobar arteries (C, D) isolated from untreated Sham (*N* = 5–7) or untreated RUPP (*N* = 4–6) rats at GD 19. Individual data points in curves statistically analyzed by a two‐way ANOVA with repeated measures followed by Sidak's multiple comparisons test. *P < 0.05 for RUPP versus Sham rats.

As our in vivo study utilized combined *α*‐ and *β*‐ adrenergic receptor blockade, we then assessed *α*_1_‐adrenergic receptor‐induced vasoconstriction in ex vivo studies. Although others have examined vasoconstriction to adrenergic agonists using various systemic arteries in RUPP rats (Zhu et al. 2016), we performed vasoconstriction studies in arteries both isolated from the systemic circulation and within the kidney, which is a player in the chronic control of blood pressure (Hall et al. 1996). Figure 4 depicts vasoconstriction responses to the *α*_1_‐adrenergic receptor agonist, Phe in a subset of untreated Sham or RUPP rats. RUPP‐induced hypertension (124 ± 6 mmHg) compared to Sham‐operated normal pregnant control rats (106 ± 4 mmHg). The rise in blood pressure was associated with greater responses to Phe‐induced vasoconstriction in third‐order mesenteric arteries (Fig. 4A). This included greater constriction values beginning at 3E^−6^M of Phe along with higher *E*_max_ (% of KCl: 263 ± 21 vs. 162 ± 32, *P *< 0.05) and AUC (310 ± 41 vs. 150 ± 30, *P* < 0.05) values in RUPP versus Sham groups. The KCl‐induced vasoconstriction curve is depicted in Figure 4B. *E*_max_ (% increase in force: 143 ± 17 vs. 201 ± 26) and AUC (10508 ± 1094 vs. 15934 ± 2125, *P* < 0.05) values in response to KCl were lower beginning at 50 mmol/L in mesenteric arteries isolated from RUPP rats. In renal interlobar arteries, vasoconstriction to Phe (Fig. 4C) was greater in RUPP over Sham rats starting at 1E^−6^M of Phe with both *E*_max_ (311 ± 19 vs. 251 ± 12, *P* < 0.05) and AUC (536 ± 34 vs. 424 ± 42, *P* < 0.05) being greater in RUPP over Sham rats. The KCl response in the interlobar arteries was similar between RUPP and Sham groups (Fig. 4D).

## Discussion

This study explored the importance of adrenergic receptor signaling in mediating placental ischemia‐induced hypertension. Placental ischemia is a major theory in the pathogenesis of PE. For this study, placental ischemia was induced by RUPP in rats, which produces hypertension. We examined the involvement of adrenergic receptor signaling in mediating RUPP hypertension. Sham or RUPP rats were administered a combination of *α*_1_‐ and *β*‐adrenergic receptor blockers. Although the treatment protocol slightly reduced blood pressure in Sham rats, this antihypertensive effect was more pronounced in the RUPP rats. Furthermore, RUPP rats had greater *α*_1_‐adrenergic receptor‐induced vasoconstriction in systemic and intrarenal arteries than Sham rats. Therefore, by utilizing an experimental model of PE, our findings suggest that adrenergic receptor signaling mediates the development of hypertension in PE.

The impetus for conducting these studies stems from the human literature where numerous reports indicate that SNA is increased in PE over normal pregnant women. These data include indirect measurements that range from no change to greater circulating catecholamine levels (Pedersen et al. 1983; Nisell et al. 1985b; Oian et al. 1986; Manyonda et al. 1998) and abnormal heart rate variability (Yang et al. 2000; Musa et al. 2016; Weber et al. 2017). More direct measurements of muscle MSNA show increased burst incidence by the third trimester in PE (Reyes et al. 2018b). However, there is relatively little information available regarding whether adrenergic receptor‐induced vasoconstriction is altered in isolated blood vessels from women with PE or intervention studies exploring the role of adrenergic receptor signaling in the pathogenesis of cardiovascular dysfunction and hypertension in this maternal disorder. The novelty of our present investigation lies in the use of in vivo and ex vivo studies to examine the role of adrenergic receptor signaling in control of blood pressure regulation and vascular tone in an experimental animal model of PE.

We examined whether adrenergic receptor signaling mediates placental ischemia‐induced hypertension. RUPP and Sham rats were treated with a combination of adrenergic receptor blockers from the time of surgery at gestational day 14 and blood pressure measurements conducted at day 19. The purpose for using combined adrenergic blockers in this study was to assess the overall impact of adrenergic receptor signaling on blood pressure regulation during pregnancy and placental ischemia‐induced hypertension. Foremost, we found a slight reduction in pressure levels in normal pregnant rats following this treatment protocol. These data are important to highlight, as studies in humans report that indirect and direct measures of SNA are increased in normal pregnant versus non‐pregnant counterparts (Usselman et al. 2015; Reyes et al. 2018a). This increase occurs early and persists throughout pregnancy, even though adrenergic vasoconstriction and blood pressure falls by term (Landau et al. 2002; Matsuo et al. 2007; Jarvis et al. 2012; Macdonald‐Wallis et al. 2012). Even with this reduction in blood pressure, acute blockade studies in animal models during normal pregnancy suggest that SNA outflow plays a role in maintenance of blood pressure (Heesch and Rogers 1995; Shi et al. 2015). For instance, acute infusions of adrenergic agonists into pregnant ewes indicate that the maternal vasculature is responsive to adrenergic receptor‐induced vasoconstriction, although this is blunted compared to nonpregnant responses (Magness and Rosenfeld 1986). Moreover, the acute hypertensive response to norepinephrine in gravid baboons is significantly attenuated by the administration of the nonselective adrenergic receptor antagonist, labetalol (Morgan et al. 1993). However, as these were acute studies, it was not clear whether adrenergic receptor signaling plays a role in chronic blood pressure control during normal pregnancy. Our present data suggest that adrenergic receptor signaling plays a role in chronic blood pressure regulation during normal pregnancy.

In comparison to normal pregnant women, SNA is further elevated in PE (Reyes et al. 2018a). Therefore, we predicted that adrenergic receptor blockade would attenuate blood pressure levels to a greater extent in RUPP rats over Sham rats. Indeed, this was observed, which implicates increased adrenergic receptor signaling in the pathogenesis of placental ischemia‐induced hypertension and PE. In contrast, there was no specific interaction between placental ischemia‐induced hypertension and adrenergic receptor blockade on heart rate. Heart rate was similarly reduced in both Sham and RUPP rats during treatment with combined adrenergic receptor blockade. These data suggest that *β*‐adrenergic receptors and their control on heart rate do not mediate the greater blood pressure fall in response to combined *α*‐ and *β*‐blockade in the RUPP rats over the Sham controls. On the other hand, this finding points to greater *α*_1_‐adrenergeric receptor signaling and vasoconstriction in mediating placental ischemia‐induced hypertension.

Studies in humans suggest that PE is a vasoconstrictive state, even more so than in gestational hypertension (Spasojevic et al. 2005). In experimental models of PE, like the RUPP rat, there is evidence that hypertension is associated with greater adrenergic receptor‐induced vasoconstriction (Brennan et al. 2016). Although others have demonstrated increased adrenergic receptor‐induced vasoconstriction in systemic arteries like small mesenteric arteries, such responses in intrarenal arteries have not been examined in PE. We believe that examining both systemic and intrarenal circulations are necessary as they each contribute to blood pressure regulation. Our current data demonstrate that adrenergic receptor‐induced vasoconstriction is greater in systemic and intrarenal arteries from RUPP compared to Sham, normal pregnant control rats. There was increased responsiveness to the *α*_1_‐adrenergeric receptor agonist, Phe, in small mesenteric arteries from RUPP over the Sham rats. However, this was accompanied by a lesser response to KCl in the RUPP versus Sham rats. These data suggest that the ratio of adrenergic receptor‐induced vasoconstriction versus KCl is greater in the mesenteric circulation from RUPP rats. Thus, it seems as if dams with placental ischemia compensate for vascular dysfunction in this circulation by induction of depolarizing mechanisms within vascular smooth muscle cells to counteract adrenergic signaling. In contrast, there is increased adrenergic receptor constriction in renal interlobar arteries without alterations in KCl‐induced vasoconstriction in RUPP rats. Overall, these ex vivo adrenergic receptor agonist experiments suggest in this animal model of PE that the hypertensive‐lowering response to combined adrenergic receptor blockade is mediated in part by reduced *α*_1_‐adrenergeric receptor‐induced vasoconstriction.

There are several mechanisms whereby increased adrenergic receptor signaling and vasoconstriction may play a hypertensive role during PE. Studies have shown that placental ischemia/hypoxia elicits the release of prohypertensive factors and reduces production of vasoprotective factors in the maternal circulation, which are implicated in promoting the hypertensive response (Maynard et al. 2003; Palei et al. 2013; Spradley et al. 2016). For example, acute hypoxia reduces levels of PlGF in placental cells (Ahmed et al. 2000). In this study, we detected falls in circulating PlGF levels in RUPP compared to Sham rats. Angiogenic factors, like PlGF and VEGF, are important for maintenance of maternal vascular health (Krysiak et al. 2005; Chau et al. 2017). PlGF plays an increased role in promoting vasorelaxation during normal pregnancy via action on its receptor, VEGFR1 (Osol et al. 2008), whereas VEGF isoforms largely bind to VEGF2 to induce relaxation (Itoh et al. 2002; Clegg and Mac Gabhann 2017). Reduced bioavailability of PlGF is a predictor of PE in humans (Zeisler et al. 2016). In pregnant mice with reduced angiogenic signaling, there is increased sensitivity to vasoconstrictors, like Phe, in mesenteric arteries (Burke et al. 2016). It was previously found that administration of recombinant human PlGF into RUPP rats prevents the development of hypertension and reduces Phe‐induced constriction (Zhu et al. 2016). In this study, adrenergic receptor blockade did not alter the reduced level of PlGF in RUPP rats but did significantly prevent their hypertension. Therefore, we hypothesize that, even in the face of the placental ischemic milieu, that adrenergic blockade protects against vascular dysfunction, exaggerated vasoconstriction, and hypertension.

While we found that placental ischemia‐induced hypertension is mediated by adrenergic receptor signaling in RUPP rats, there are limitations to this study. Foremost, this study was not designed to assess whether SNA is increased in this model of PE. We did not examine SNA, but others found changes in homeostatic mechanisms that regulate this activity, including baroreceptor and chemoreceptor reflexes. There have been several studies showing alterations in these reflexes on control of cardiovascular function during PE. Human studies have demonstrated variable levels of baroreflex function. Some studies show increased blood pressure variability but unaltered baroreceptor control of heart rate (Faber et al. 2004), whereas others detected reductions in baroreflex control of blood pressure (Wasserstrum et al. 1989; Silver et al. 2001) and heart rate (Molino et al. 1999) or increases in baroreceptor control of heart rate (Weber et al. 2017). However, these studies were only associational in design and have not examined mechanisms whereby these changes occur. For example, whether these alterations are associated with the extent of placental ischemic disease. RUPP rats with placental ischemia have blunted baroreflex gain, but this was similar to normal pregnant controls; however, there was a hypertensive shift in the function of this system (Hines et al. 2007). There have been no direct intervention studies relating changes in baroreflex function in mediating or maintaining the hypertension in women or experimental animals with PE. No such studies exist for chemoreflex function or oxygen sensing either. There is an association between sleep‐disordered breathing, vascular dysfunction, and PE in humans (Yinon et al. 2006). Experimental models show that exposure to chamber hypoxia promotes increased circulating placental ischemic factors and maternal vascular dysfunction and hypertension (Lai et al. 2011; Aljunaidy et al. 2016). However, the relative contribution of hypoxia‐induced activation of chemoreceptors and their promotion of SNA and adrenergic receptor signaling in mediating hypertension in PE should be examined. Future studies should research whether blocking central mechanisms of sympathetic drive to cardiovascular‐renal tissues mediates the development of placental ischemia‐induced hypertension. This includes utilizing biotelemetry technology in pregnant animals to continually measure blood pressure before and after the RUPP procedure and during selective blockade of central SNA pathways or peripheral adrenergic receptors in normal pregnant and RUPP groups. Also, in regards to the central nervous system, it has yet to be established whether placental ischemic factors, like those antiangiogenic in nature, have a direct impact on sympathetic drive and control of blood pressure. However, another factor released in response to placental ischemia, namely tumor necrosis factor (TNF)‐*α*, has been shown in nonpregnant hypertensive models to cross the blood–brain barrier and stimulate sympathetic outflow to organs like the kidney and induce hypertension (Zhang et al. 2003; Purkayastha et al. 2011). Although it is established that TNF‐*α* infusion produces hypertension in once normotensive pregnant rats (Alexander et al. 2002), the relative role of central pathways versus peripheral vascular dysfunction and adrenergic receptor‐induced vasoconstriction in this hypertensive response during pregnancy should be determined. Subsequent studies will address individual receptor expression via radioligand binding and their relative control of blood pressure and vascular function by examining isolated blood vessels from animals treated with individual blockers of the different adrenergic receptor subtypes in normal pregnant versus PE models, like RUPP, or chronic infusion of placental ischemic factors.

## Significance and Perspectives

Although human and experimental animal studies have demonstrated increased SNA and adrenergic receptor‐induced vasoconstriction during PE, these were only association studies (Reyes et al. 2018a). Before now, there was a lack of intervention studies examining the cause‐and‐effect relationship between adrenergic receptor signaling and the pathogenesis of hypertension in PE. Our study is one of the first to demonstrate that chronic adrenergic receptor blockade attenuates the development of hypertension in an experimental model of PE. This protocol utilized an animal model of placental ischemia‐induced hypertension. Placental ischemia is a major theory in the pathogenesis of PE (Weel et al. 2016). In this study, rats were chronically administered adrenergic receptor blockade. As this protocol attenuated placental ischemia‐induced hypertension more so than the blood pressure fall in normal pregnant rats, these data suggest that placental ischemia exaggerates adrenergic receptor signaling in the maternal vasculature to mediate the hypertension. This study opens the door for additional research directed at better understanding mechanisms promoting elevations in maternal SNA and adrenergic receptor signaling during hypertensive disorders of pregnancy.

## Conflict of Interest

None declared.
